# Circular RNAs: A novel type of biomarker and genetic tools in cancer

**DOI:** 10.18632/oncotarget.18350

**Published:** 2017-06-02

**Authors:** Yi-Neng Han, Sheng-Qiang Xia, Yuan-Yuan Zhang, Jun-Hua Zheng, Wei Li

**Affiliations:** ^1^ Department of Stomatology, Shanghai Tenth People's Hospital, Tongji University, Shanghai, 200072, China; ^2^ Odense University Hospital, Department of Nephrology, University of Southern Denmark, Institute for Molecular Medicine, Cardiovascular and Renal Research Institute of Clinical Research, Odense, 999017, Denmark; ^3^ Wake Forest Institute For Regenerative Medicine, Wake Forest University, Winston-Salem, NC, 27103, USA; ^4^ Department of Urology, Shanghai Tenth People's Hospital, Tongji University, Shanghai, 200072, China; ^5^ Department of Urology, Shanghai Jiao Tong University Affiliated First People's Hospital, Shanghai, 200080, China

**Keywords:** circular RNAs, cancer, microRNA sponges, gene transcription, biomarkers

## Abstract

Circular RNAs (circRNAs) are a novel type of universal and diverse endogenous noncoding RNAs (ncRNAs) and they form a covalently closed continuous loop without 5′ or 3′ tails unlike linear RNAs. Most circRNAs are presented with characteristics of abundance, stability, conservatism, and often exhibiting tissue/developmental-stage-specific expression. CircRNAs are generated either from exons or introns by back splicing or lariat introns. CircRNAs play important roles as miRNA sponges, gene transcription and expression regulators, RNA-binding protein (RBP) sponges and protein/peptide translators. Emerging evidence revealed the function of circRNAs in cancer and may potentially serve as a required novel biomarker and therapeutic target for cancer treatment. In this review, we discuss about the origins, characteristics and functions of circRNA and how they work as miRNA sponges, gene transcription and expression regulators, RBP sponges in cancer as well as current research methods of circRNAs, providing evidence for the significance of circRNAs in cancer diagnosis and clinical treatment.

## INTRODUCTION

Over 70% of the human genome is found actively transcribed, but protein-coding genes only account for 1∼2% of the human genome, whereas the vast majority of transcripts are noncoding RNAs (ncRNAs) [1]. ncRNA can be divided into two major groups, including housekeeper ncRNAs, namely ribosomal RNA (rRNA), transfer RNA (tRNA), small nuclear RNA (snRNA) and small nucleolar RNA (snoRNA), as well as regulatory ncRNAs. Regulatory ncRNAs can be categorized according to the length, including small noncoding RNAs with transcripts shorter than 200 nucleotides like microRNAs (miRNAs), snRNAs, piwi-interacting RNA (piRNAs), small interfering RNA (siRNAs) and others, as well as long noncoding RNAs (lncRNAs) whose transcripts longer than 200 nucleotides [2]. Circular RNAs (circRNAs) are a naturally occurring class of noncoding RNAs having been paid more and more attention. They form a covalently closed continuous loop without 5′ caps and 3′ tails, which make themselves resistant to RNase R activity and more stable than liner RNAs [3]. It was more than two decades ago that circRNA was first found by Nigro et al. [4], but it was thought to be molecular flukes or artifacts of aberrant RNA splicing with no functions [5]. Only recently, with the rapid development of high-throughput RNA sequencing (RNA-Seq) technology and bioinformatics method, circRNAs are more and more extensively investigated and their important roles as miRNA sponges [2, 6, 7], gene transcription and expression regulators [7, 8], and RNA-binding proteins (RBP) sponges [9–11] are uncovered. Furthermore, numerous of studies have confirmed the function of circRNAs in tumor cell proliferation, migration and invasion, which may potentially serve as a required novel biomarker and therapeutic target for cancer treatment [12–14].

In this review, we will first discuss about the origins, characteristics and functions of circRNA. Then we talk about how they work as miRNA sponges, gene transcription and expression regulators, RBP sponges in cancer and have a briefly introduction of current research methods of circRNAs in cancer, providing evidence for the significance of circRNAs in cancer diagnosis and clinical treatment.

### Origins and public databases of circRNAs

**Figure 1:**
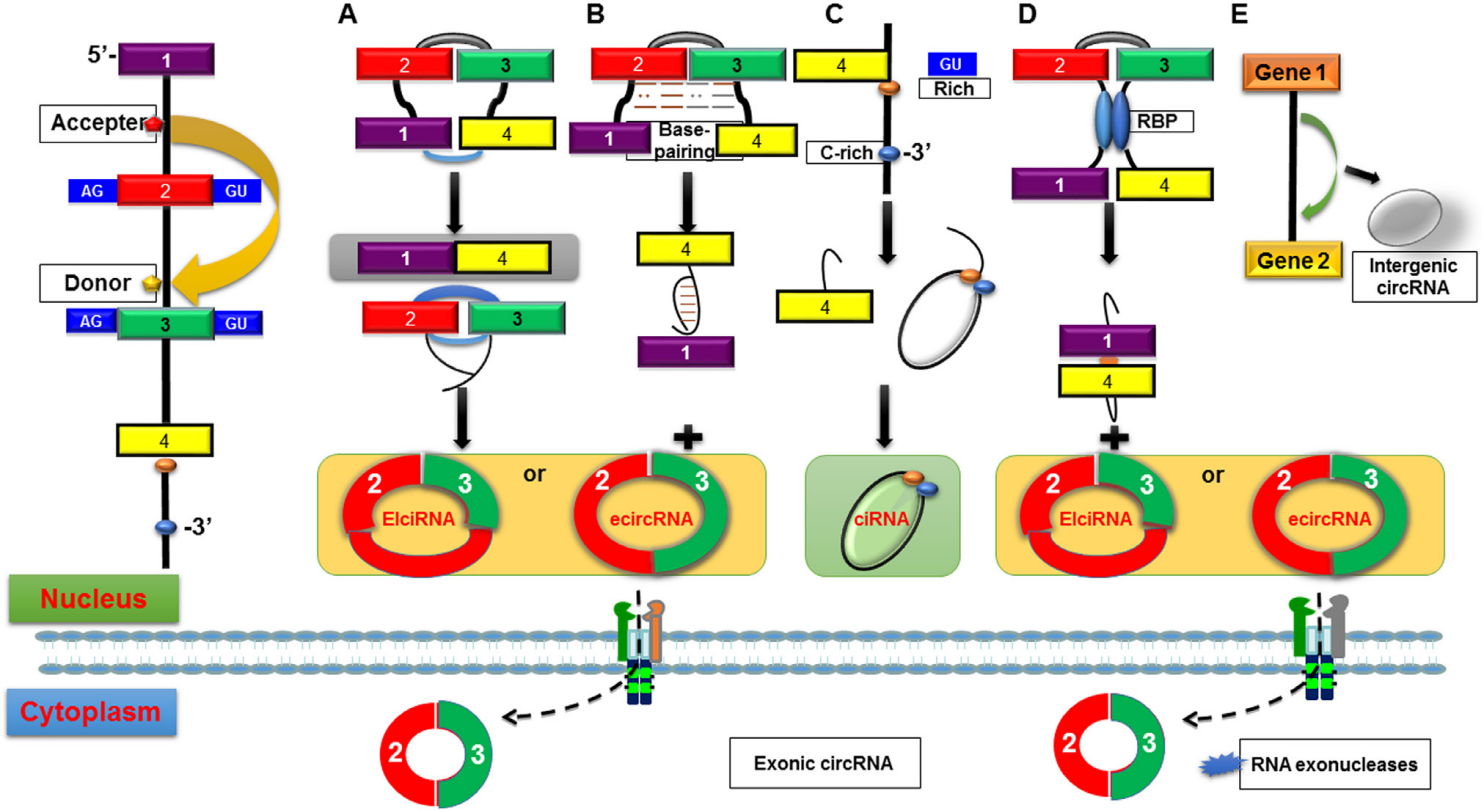
Possible biogenesis patterns of circRNAs (**A**) Lariat-driven circularization. ecircRNA or EIciRNA are generated by exon skipping event where 5′ exon attacks 3′ splice site, forming a lariat structure containing the skipped exons 2 and 3 as well as an mRNA consisting of exons 1 and 4. (**B**) Intron-pairing-driven circularization. circRNA or EIciRNA are formed by removed or retained introns from direct base-pairing of the introns flanking inverted repeats or ALU elements. (**C**) ciRNAs. ciRNAs lies on a consensus motif containing a GU-rich element (red dot) near the 5′ splice site and an C-rich element (blue dot) near the branchpoint site, which are capable for an intron to escape debranching and become a stable circRNA. (**D**) circRNA biogenesis depends on RBPs. Serving as a bridge linking two flanking introns close together, RBPs are capable to promote the formation of circRNAs by binding to sequence motifs of flanking introns. A circRNA or an EIciRNA is generated due to the retention of internal introns. (**E**) Intergenic circRNA. Because of their resistance to RNase R, ecircRNAs are extraordinary stable and are always located in the cytoplasm, while ciRNAs and EIciRNAs are predominately located in the nucleus.

There are mainly three categories of circRNAs according to multiple biogenesis patterns (Figure 1): exonic circRNAs (ecircRNAs) [7], retained-intron circRNAs or ElciRNAs [8] and circular intronic RNAs (ciRNAs) [9]. It has been reported that circRNAs are generated by both canonical and noncanonical splicing, which are quite different from the canonical splicing linear RNAs. Among circRNAs, ecircRNAs are the most, accounting for more than 80% of identified circRNAs. Jeck et al. [7] first put forward two models of ecircRNA formation: lariat-driven circularization and intron-pairing-driven circularization. Emerging evidence has revealed that circRNA molecules are mainly generated by a process called back-splicing, where downstream exons are spliced to upstream exons in reverse order [15]. That is to say, a splice donor site joins to a splice acceptor site upstream in the primary transcript, producing a circular transcript. It is believed that exon circularization depends on flanking intronic complementary sequences and alternative formation of inverted repeated Alu pairs which may help determine the production rate of circRNAs [15]. Usually, introns between the encircled exons are spliced out, but in some circumstances, they are retained which called retained-intron circRNAs or ElciRNAs [10]. The origine of ciRNAs lies on a consensus motif containing a 7 nucleotide (nt) GU-rich element near the 5′ splice site and an 11 nt C-rich element near the branchpoint site.

Besides, there is another model of circRNA biogenesis depend on RBPs. Splicing factors Quaking (QKI) [17] and Muscleblind (MBL) [18], served as a bridge linking two flanking introns close together, are capable to promote the formation of circRNAs. On the contrary, Li et al. [10] reported that RNA-editing enzyme ADAR1 could abolish circRNA formation by binding to double-stranded RNA to melt the stem structure. These findings indicate that RBPs play critical roles in activating circularization by bridging complementary sequences and inhibiting canonical splicing. However, further researches are required to elucidate the detailed mechanisms of circRNAs biogenesis.

Currently, there are several online databases (Supplementary Table 1) which collect circRNAs from GenBank annotations or published articles. Some ncRNAs in the database have been experimentally proved, some are purely computational predictions and some are annotated as ncRNAs based on the open reading frame (ORF) predicted size. These databases make us better investigate and understand circRNAs and their association with diseases.

### Characteristics of circRNAs

Emerging evidence revealed several highlighted characteristics of circRNAs: 1) Abundance: Salzman et al. [27] first put forth that circRNAs are the most universal molecules exceed that of related linear mRNAs distributed in human cells; 2) Stability: CircRNAs presented with more stable property than linear mRNAs due to their covalently closed loop structures which confer them resistant to RNase R [27]. It is reported that the average half-life of circRNAs in most species is more than 48 h, while the half-life of mRNAs on average is about 10 h [28]; 3) Conservatism: circRNAs are highly conserved in different species. For example, many circRNAs can be detected in both humans and mice including Drosophila [7, 29]; 4) Location: ecircRNAs making up majority of circRNAs are predominantly cytoplasmic and probably possess miRNA response elements (MREs) [2, 30]. Intronic circRNAs, namely ciRNAs and EIciRNAs, are primarily located in the nucleus in eukaryotes and may take part in gene expression regulation at the transcription or post-transcription level [7, 9]; 5) CircRNAs often exhibit tissue/developmental-stage specific expression. For example, circRNAs expressed high in the mammalian brain, especially in the synapses, and during neuronal differentiation circRNAs are dynamically up-regulated [31]; 6) Some circRNAs contain miRNA binding sites and can competitively attenuate endogenous miRNA-mediated activities [7].

### Functions of circRNAs

**Figure 2:**
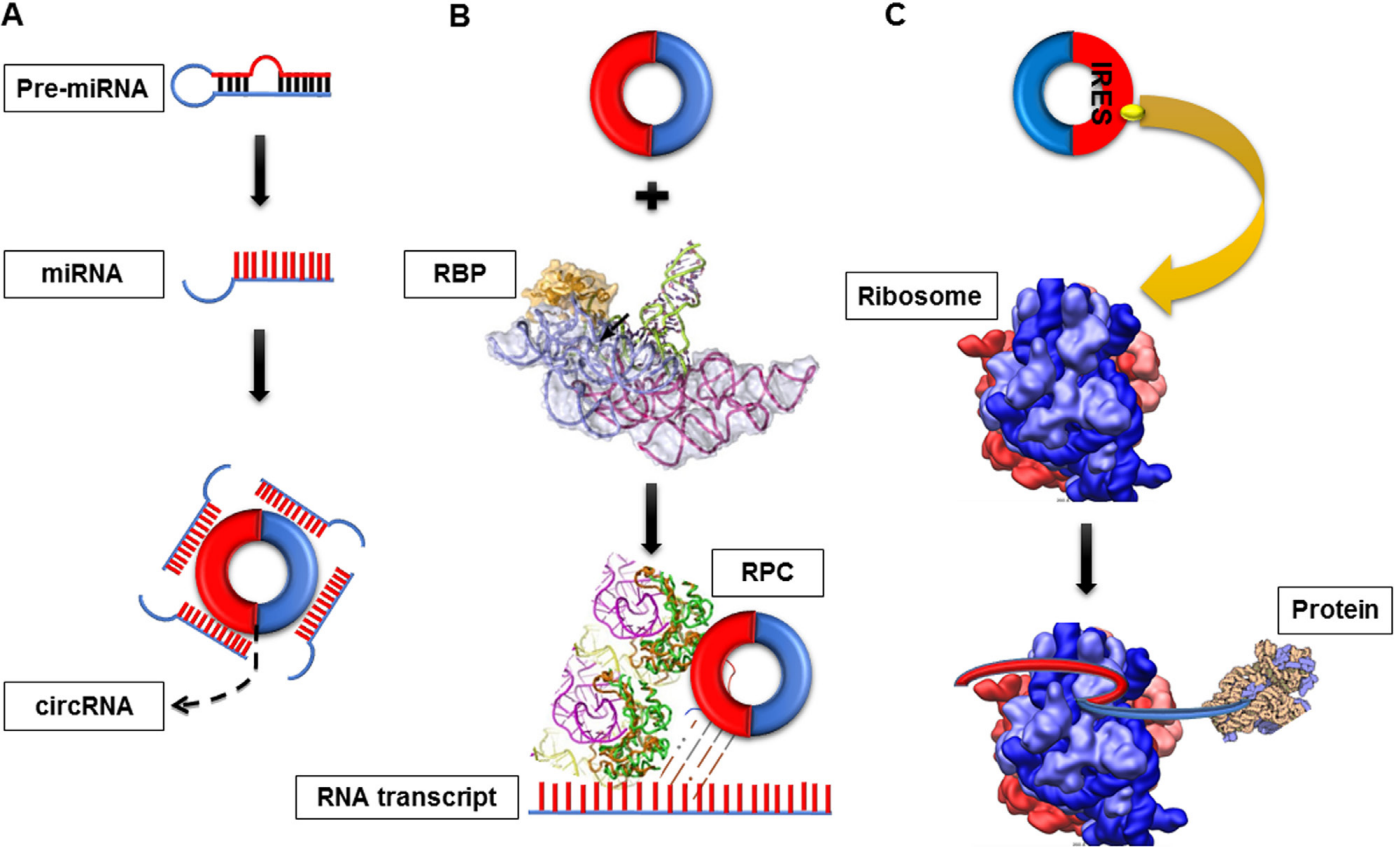
Functions of circRNA (**A**) CircRNA as a miRNA sponges. CircRNAs can negatively regulate miRNAs activities by competing for miRNA-binding sites (**B**) CircRNA as RNA-binding protein (RBP) sponges. CircRNAs can bind RPBs to form RNA-protein complex (RPC) and then interact with the linear transcript of gene. (**C**) CircRNAs as protein translators. circRNAs which contain internal ribosome entry site elements (IRES) or prokaryotic ribosome-binding sites could generate functional proteins/peptide.

CircRNAs can serve as miRNA sponges, gene transcription and expression regulators, RBP sponges and protein/peptide translators by affecting the gene expression level from the transcription or post-transcription level (Figure 2).

### CircRNAs as miRNA sponges

As endogenous RNAs (ceRNAs), circRNAs can negatively regulate miRNAs activities by competing for miRNA-binding sites [2, 6]. The most representative is the human circRNA cerebellar degeneration-related protein 1 transcript (CDR1as) / ciRS-7, which contains 74 selectively conserved miRNA target sites, serving as miR-7 sponge. ciRS-7 originates from the transcript antisense to the CDR1 gene and is sensitive to miR-671, which can be endonucleolytically cleaved via binding to miR-671 in an miR-7 target site Argonaute2 (AGO2)-dependent manner. Therefore, by cleavage of ciRS-7, miR-7 is transported to a subcellular location where miR-7 is released by miR-671 activity. It has been confirmed that overexpression of circRNA CDR1 /ciRS-7 increased the expression of miRNA target genes, while knockdown of it has an opposite effect. Moreover, the sex-determining region Y (Sry) and the testis-specific circRNA, serves as the miR-138 sponge [6]. At present, acting as miRNA sponges is the main function of some circRNAs.

### CircRNAs as gene transcription and expression regulators

Accumulating evidence has revealed that circRNAs play a pivotal role in post-transcriptional and gene expression regulation. It is shown that EIciRNAs such as circEIF3J, circPAIP2 are mainly localized in the nucleus, interacting with U1 small nuclear ribonucleoprotein particle (U1 snRNP) and RNA polymerase II (Pol II) to enhance the transcription of their parental genes [10, 32].

Additionally, circular intronic RNA (ciRNA) ci-ankrd52 is related with elongation Pol II machinery and positively regulates Pol II transcription by largely accumulating to its transcription sites. Knockdown of ci-ankrd52 could reduce the expression of their parental genes [9]. These data suggests that EIciRNAs and ciRNAs may regulate transcription in the nucleus while exonic ecircRNAs may possibly serve as miRNA sponges in the cytoplasm.

### CircRNAs as RBP sponges

RBPs are involved in a variety of bioactivities such as cell proliferation, differentiation, motility, apoptosis, senescence and cellular responses to oxidative stress through post-transcriptional regulation like RNA alternative splicing, stability, transport and translation [33, 34]. Previous studies indicated that circRNAs could serve as RBP sponges by stably associated with Argonaute (AGO) proteins [6], RNA QKI [17], MBL [18], Pol II [9], eukaryotic initiation factor 4A-III (EIF4A3) [35] and so on to form large RNA-protein complexes (RPCs). These RPCs could regulate the pool of RBPs or small RNAs and then interact with the liner RNA counterparts [27]. Besides, through analysis of RBP binding sites on human circRNAs by a new web tool, CircInteractome, exceptionally high density of binding sites of circRNAs for a given RBP are found [22]. For example, hsa_circ_0024707 acts as a sponge for AGO2 with 85 predicted positions, and the mature hsa_circ_0000020 contains several RBPs binding sites such as FMRP (10 sites) and HuR (6 sites) [35].

### CircRNAs as protein/peptide translators

It was reported that some circRNAs containing internal ribosome entry site elements (IRES) [36] or prokaryotic ribosome-binding sites [32] could encode proteins unlike their canonical counterparts. There is a circRNA database, named circRNADb, containing 32,914 human exonic circRNAs which may offer detailed information of the circRNAs, including genome sequence, ORF and IRES to users for prediction of the translatability of certain circRNAs [37]. Currently, Yang et al. [38] first reported that N^6^-methyladenosine (m^6^A), the most abundant base modification of RNA, promotes efficient initiation of protein translation from circRNAs in human cells. They discovered that consensus m^6^A motifs are enriched in circRNAs and a single m^6^A site is sufficient to drive translation initiation. The m^6^A-driven translation is initiated by eIF4G2 and m6A reader YTHDF3, and is enhanced by methyltransferase METTL3/14, inhibited by demethylase FTO, and up-regulated upon heat shock. Furthermore, m^6^A-driven translation of circRNAs is revealed to be widespread and hundreds of endogenous circRNAs have translation potential, suggesting a role of circRNA-derived proteins in cellular responses to environmental stress.

### CircRNAs in cancer

Accumulating evidence has revealed that circRNAs are correlated with various human diseases such as atherosclerosis [39], Alzheimer's disease [40], Parkinson's disease, diabetes [41] and especially cancers. Numerous investigations have been carried out to explore the aberrant circRNAs expressions in tumorogenesis, which may be considered as diagnostic and therapeutic biomarkers for cancer (Supplementary Table 2).

### CircRNAs as miRNA sponges in cancer

The role of circRNAs as a miRNA sponge is the main mechanism of circRNAs in cancer. The most well-known miRNA sponge is CDR1as/ciRS-7, which comprises more than 70 miR-7 binding sites and serves as a miR-7 sponge. Accumulating evidence has revealed that miR-7 acts as a tumor suppressor in a variety of cancers, such as hepatocellular carcinoma (HCC) [42], gastric cancer (GC) [43], colorectal cancer (CRC) [44], breast cancer [45], cervical cancer [46], lung neoplasm [26], tongue cancer [47], Schwannoma tumor [48]. Chou et al. [49] found that in lung cancer tumorogenesis, ciRS-7 could increase the expression of miR-7 target oncogenes and decrease the tumor suppression genes to significantly reduce the activity of miR-7. Many cancer-related pathways have been involved in miR-7-associated regulations, such as directly down-regulated oncogenic factors epidermal growth factor receptor (EGFR) [50], mammalian target of rapamycin (mTOR) [42], Yin Yang 1 (YY1) [44], insulin receptor substrate-1 (IRS-1) and insulin receptor substrate-2 (IRS-2) [50], phosphoinositide 3-kinase catalytic subunit delta (PIK3CD) [51], Raf1 [51], Ack1 [48], P21-activated kinase-1 (Pak1) [52] and PA28γ [53]. Moreover, miR-7 indirectly down-regulates signal transducer and activator of transcription3 (STAT3) by down-regulating histone-lysine N-methyltransferase1 (SETDB1), resulting in epithelial to mesenchyme transition (EMT), suppressing breast cancer stem cells invasion and metastasis [54]. Ning et al [55] found that miR-7 directly targets and attenuates p65 and then activates NF-κB to inhibit HCC metastasis. ciRS-7 acts as a risk factor of hepatic microvascular invasion in HCC [56].

But on the contrary, Honegger et al. [57] presented that viral oncogene E6/E7 is related to miR-7 overexpression in HPV-positive HeLa cell line and Nakagawa et al. [58] revealed miR-7 expression is increased in advanced colorectal cancers. These examples demonstrate that the miR-7/miR-671/ciRS-7 axis possibly participates in cancer-associated biological processes by either serve as a cancer suppressor or promoter largely relied on the expression level of miRNA target genes.

Additionally, cir-ITCH as sponges of miR-7 and miR-20a is involved in colorectal cancer [59]. And cir-ITCH as miR-7, miR-17 and miR-214 sponges may have a tumor suppressive role in esophageal squamous cell carcinoma (ESCC) [12] while cir-ITCH as miR-7 and miR-214 sponges takes part in lung cancer. It has shown that the role of cir-ITCH in tumor formation and chemosensitivity through miRNA regulation is mediated by Wnt/β-catenin signaling pathway via ubiquitination and degradation of phosphorylated Dvl2 [59, 60]. Another circHIPK3 has been identified to be associated with cancer progression by binding miR-124, which exhibited the most prominent binding effect [61]. Foxo3 circular RNA (circ-Foxo3) increases Foxo3 translation and further suppresses tumor growth, cancer cell proliferation and survival by acting as a sponge of potential miRNAs [62]. CircRNA_1093, which contains 4 miR-342-3p binding sites, is implicated in BRCA1 expression in breast cancer [63, 64]. CircZEB-1.17, circ-ZEB1.19, circZEB1.33 and circZEB1.5 are down-regulated in the lung cancer as the miR-200 sponge [21]. Hsa_circ_001569 positively regulates cell proliferation and invasion of colorectal cancer by acting as a miR-145 sponge. It inhibits the transcriptional activity of miR-145 without influencing its expression level, resulting in the upregulation of target genes of miR-145, including E2F5, BAG4, and FMNL2 [13].

Currently, Zheng et al. [65] found that circ-TTBK2 was up-regulated in glioma tissues and cell lines acted as miR-217 sponge in a sequence-specific manner but not linear TTBK2. In addition, upregulated circ-TTBK2 decreased miR-217 expression and there was a reciprocal negative feedback between them in an Argonaute2 (AGO2)-dependent manner. As miRNA sponges, circRNAs provide us a novel insight of cancer treatment, but there remain quantities of unknown circRNAs and specific circRNA-miRNA-gene regulatory mechanisms in cancer initiation and progression which need further investigations.

### CircRNAs as transcription regulators in cancer

As mentioned above, certain circRNAs like ci-mcm5 and ci-sirt7 can enhance the transcription of their parental genes which suppress or promote cancer progression [9, 66]. For instance, overexpression of MCM5 is believed to be involved in both colorectal cancer and oral squamous cell carcinoma, predicating poor outcomes [67, 68]. Inversely, low expression of SIRT7 is related to aggressive tumor phenotype and poor prognosis in pancreatic ductal adenocarcinoma (PDAC) [69]. cZNF292 is found to suppress glioma cells proliferation and vascularization by reducing the expression of cellular Cyclin A, p-CDK2, CDK2, β-catenin, p-STAT3(Tyr705) and p-STAT5 (Tyr694). And downregulation of cZNF292 lead to decreased transcription of E2F1, NF-κB, Sp1, HIF-1, AP-1, STAT3, and STAT5, inhibiting tube formation of tumor cells [70]. Therefore, circRNAs that are considered as a type of alternative splicing isoforms may play a key role in regulating gene expression, leading to cancer-related dysregulation.

### CircRNAs as RBP sponges in cancer

It has been revealed that RBPs such as QKI, AGO, Pol II and MBL can bind to circRNAs and RBP misregulation of gene transcription or expression plays an important role in cancer progression [71, 72]. The most widely studied is the RNA-binding protein quaking 5 (QKI-5), which has been recognized as a novel tumor suppressor in many cancers, including lung cancer and prostate cancer [73, 74]. CircRNAs themselves are regulated during epithelial-mesenchymal transition (EMT), which is involved in the progression of cancer metastasis. The up-regulation of circRNAs during EMT implies they carry out important functions in EMT [17]. But circRNAs formation is regulated by QKI proteins, providing a novel perspective to QKI-mediated circRNAs therapeutic strategies in cancer. Meanwhile, AGO proteins was also revealed to be ectopically over-expressed in cancer and closely related to the cancers development via miRNAs-dependent or independent pathways [75]. These observations suggest that circular RNAs and RBPs might interact with each other to have effect on tumorogenesis.

In addition, circ-Foxo3 plays a role in cell cycle suppressor by binding to the cell cycle proteins cyclin-dependent kinase 2 (CDK2) and cyclin-dependent kinase inhibitor 1 (or p21), forming a ternary complex to inhabit the function of CDK2 and block cell cycle progression. CDK2 could interacts with cyclin A and cyclin E to facilitate cell cycle entry, while p21 could inhibit these interactions and arrest cell cycle progression [76]. As CDK2 is associated with multiple cancers, like breast cancer [77], colorectal cancer [78], non-small cell lung cancer (NSCLC) [79], hence, circ-Foxo3 also carry out functions in cancers above by forming circ-Foxo3-p21-CDK2 ternary complex. What's more, circ-Foxo3 can also bind to proteins ID1, E2F1, FAK, and HIF-1α, retaining them in the cytoplasm and promoting cardiac senescence [76]. These findings indicate that circRNAs may take part in cancer progression by binding to partners.

Recently, Abdelmohsen et al. [80] newly identified some circRNAs binding HuR in human cervical carcinoma HeLa cells. One of the most prominent HuR target circRNAs was hsa_circ_0031288, renamed CircPABPN1 as it arises from the PABPN1 pre-mRNA. Further analyses revealed that HuR did not influence CircPABPN1 abundance while high levels of CircPABPN1 suppressed HuR binding to PABPN1 mRNA, providing the first example of competition between a circRNA and its cognate mRNA for an RBP that affects translation.

### CircRNAs as potential biomarkers in cancer

As mentioned above, circRNAs have the characteristics of abundant, conservative across species, stably expressed in saliva, blood, and exosomes, exhibiting tissue/developmental-stage specific property, which meet the requirement of promising cancer biomarkers. Besides, circRNAs are more likely to be detected than miRNAs whose number is relatively small. In addition, the way of testing circRNAs by RT-PCR and in-situ hybridization is more sensitive and specific than detecting proteins by an antigen-antibody reaction. So circRNAs may have the advantage of serving as potential cancer biomarkers. There are already various circRNAs recognized as potential biomarkers. For example, circ_100855 predominately up-regulated and circ_104912 significantly down-regulated in laryngeal squamous cell cancer tissues (LSCC) tissues, and their expressions are remarkably associated with tumor stage and neck nodal metastasis, implying a potential novel biomarker in LSCC tumorigenesis [81]. By using qRT-PCR, hsa_circ_100855 were detected as the most up-regulated circRNA and hsa_circ_104912 as the most down-regulated circRNA, implying that patients with T3-4 stage, neck nodal metastasis or advanced clinical stage had higher hsa_circ_100855 expression and lower hsa_circ_104912 expression [81]. Analogously, circ_002059 down-regulated in gastric cancer is related with distal metastasis, TNM stage, gender, and age, serving as a pivotal role for the diagnosis of gastric cancer [14]. Besides, hsa_circ_0000190 [82], hsa_circ_0000096 [83], and circPVT1 [84] are newly found to be promising gastric cancer biomarkers. Similarly, hsa_circ_0001649 [85] and hsa_circ_0005075 [86] are regarded as critical biomarkers for the physiological and pathological processes of hepatocellular carcinoma. In addition, hsa_circ_001988 [87] as well as hsa_circ_0000069 [88] is involved in colorectal cancer progression. Zhu et al.[89] recently characterized the circRNA expression profile from three paired colorectal cancer and adjacent normal tissues by human circRNA array, and validated one circRNA generated from Exon 5-11 of BANP gene, termed circ-BANP. The results demonstrated that dysregulated circ-BANP appears to have an important role in colorectal cancer cells and could serve as a prognostic and therapeutic marker for colorectal cancer. ciRS-7 was found significantly up-regulated in colorectal cancer tissues and is a promising prognostic biomarker in colorectal cancer patients, serving as a therapeutic target for reducing oncogenes EGFR-RAF1 activity [90]. Li et al. [91] examined serum exosome RNA sequencing datasets from 11 patients with colorectal cancer and normal serum, identifying that 67 circRNAs were missing as well as 257 new circRNA species were detected in colorectal cancer patients. Among the newly detected circRNAs, 48 genes for 53 circRNAs were remarkably up-regulated. Recently, circBRAF was found to be significantly down-regulated in glioma patients with high pathological grade (WHO III & IV) than those with low grade (WHO I & II) (*P* < .001). CircBRAF could severe as a biomarker for predicting pathological grade and prognosis in glioma patients [92]. What's more, hsa_circ_0067934 is demonstrated to be up-regulated in ESCC tissues and represents a novel potential biomarker and therapeutic target of ESCC [93]. In bladder cancer, overexpression of circTCF25 promotes proliferation and migration might be through circTCF25-miR-103a-3p/miR-CDK6 pathway, suggesting a new promising marker for bladder cancer [94]. Li et al. [95] used microarray to identify dysregulated circular RNAs in PDAC patients and demonstrated clusters of aberrantly expressed circRNAs in PDAC, providing new potential targets for the future treatment of PDAC and novel insights into PDAC biology. Moreover, a total of 322 circRNAs were aberrantly expressed between cutaneous squamous cell carcinoma and non-lesional skin biopsies. The same cohort also discovered 71 differentially expressed circRNAs in basal cell carcinoma [96].

Recently, well-established cancer-associated chromosomal translocations gave rise to fusion circRNAs (f-circRNA) which were produced from transcribed exons of distinct genes affected by the translocations. Guarnerio et al. [97] found that f-circRNA plays an active role in favoring leukemia progression when coupled with other oncogenic stimuli. Alhasan et al. [98] found that circRNAs are enriched in human platelets 17- to 188-fold relative to nucleated tissues because liner RNAs are more likely to be degraded. Besides, circRNAs can also participate in platelets relative miRNA regulations acting as endogenous competitive RNAs [99]. f-circM9 and f-circPR are formed by the PML/RARa and MLL genes fusion and knockouts of f-circM9 and f-circPR could lead to apoptosis of quantities of tumor cells and increases their sensitivity to drugs, such as arsenic as well, suggesting that f-circM9 and f-circPR play a oncogenic role in hematological malignancy [97]. Taken together, aberrantly expressed circRNAs in human cancers can be employed as a new class of diagnostic, prognostic and therapeutic biomarkers.

### CircRNAs with microarray in cancer

**Figure 3:**
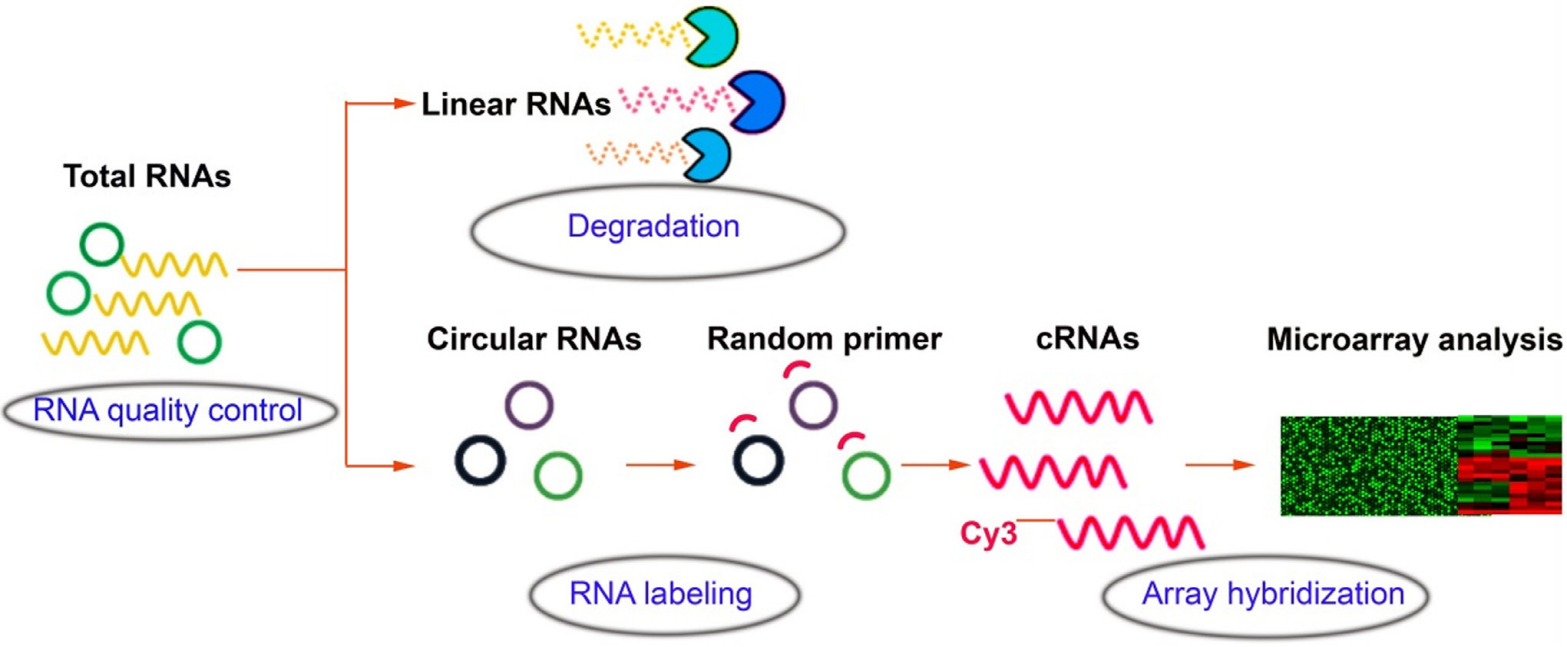
Workflow of microarray analysis of circRNAs

**Figure 4:**
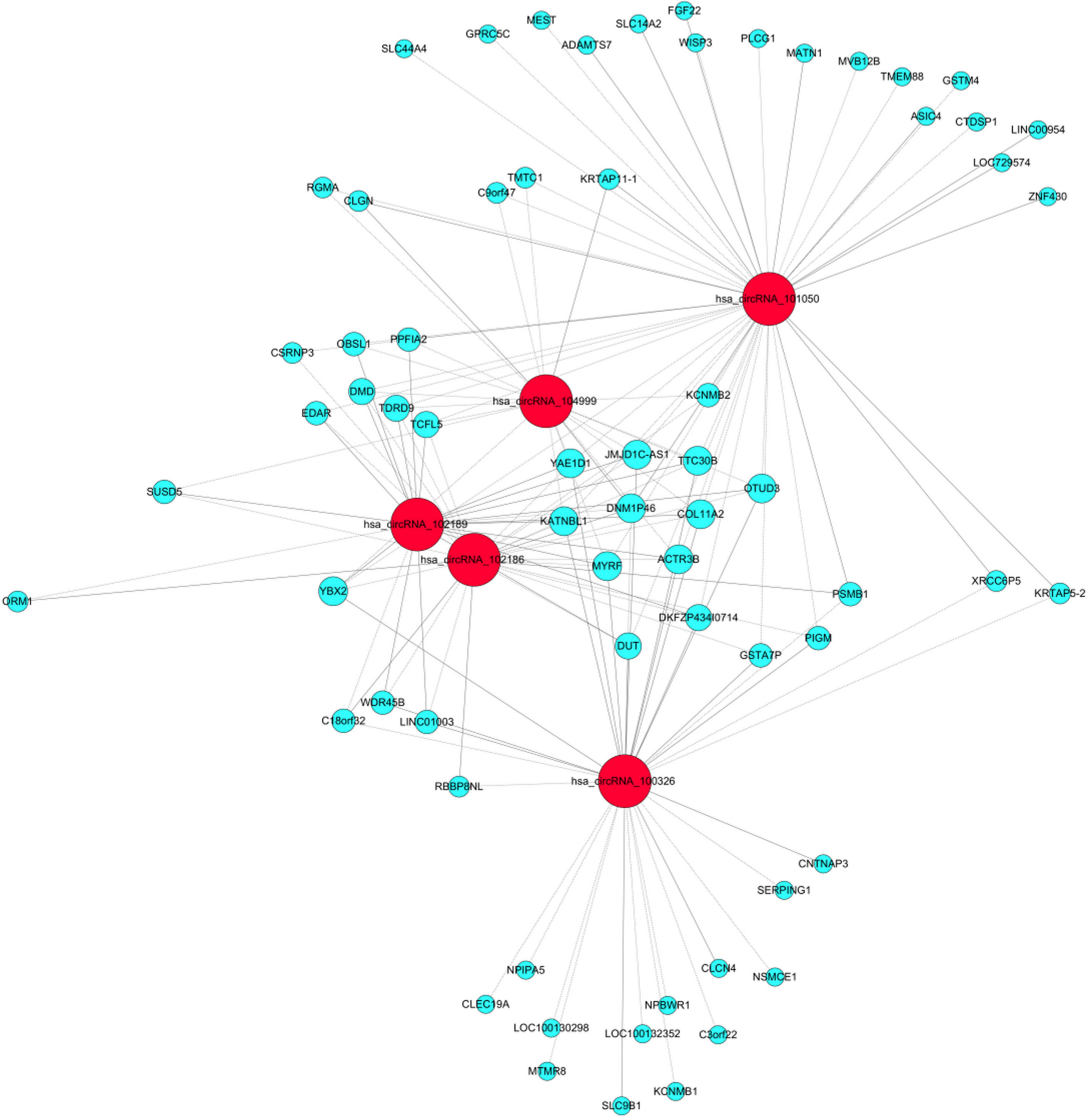
Bioinformatics analysis combined with microarray in kidney cancer

Over the past decades, the gene expression microarray has been recognized as a useful and feasible approach to profile the molecular signatures, including circRNAs. The experiment workflow of microarray expression profile of circRNAs (Figure 3) is quite different from the liner RNAs. For example, Su et al. [100] executed microarray analysis and bioinformatic tools to investigate the role of circRNAs in the radioresistance of esophageal cancer. Results showed that there was more than 400 target genes enrichment on Wnt signaling pathway and circRNA_001059 and circRNA_000167 were the two largest nodes in circRNA/microRNA co-expression network. In addition, to explore lncRNAs and circRNAs expression profiling and their biological functions in bladder cancer tumorigenesis, Huang et al. [101] used microarray to identify aberrantly expressed circRNAs, lncRNAs and mRNAs. GO and KEGG pathway enrichment analyses were executed to determine the principal functions of the significantly deregulated genes and bioinformatics methods were constructed to explore correlated expression networks including ceRNA, cis regulation, coding-noncoding co-expression (CNC), lncRNAs-transcription factor (TF)-mRNA. Results indicated that lncRNA H19 and circRNA MYL could bind competitively with miRNA-29a-3p, increasing target gene DNMT3B, VEGFA and ITGB1 expressions. Hence, lncRNAs and circRNAs could play a critical role in the pathogenesis and development of bladder cancer. Recently, Zhang et al. [102] identified 46 differently expressed circRNAs between cancer and adjacent normal tissues by using microarray. Then a four-circRNA-based classifier was constructed to evaluate the early recurrence of stage III gastric cancer after radical surgery, whose area under the receiver operator characteristic curve (ROC) reached to 0.763. Currently, microarray was used to screen differently expressed circRNAs and mRNAs in gastric cancer and adjacent tissues. CircRNAs regulating the expression of target genes through interactions with miRNAs via a variety of mechanisms might become new molecular biomarkers for gastric cancer in the future [103]. Besides, our research team used bioinformatics method combined with microarray to analysis associated circRNAs in kidney cancer (Figure 4), which owned intellectual property rights.

### CircRNA with RNA-Seq in cancer

Rapid development of high-throughput RNA sequencing (RNA-Seq) technology and bioinformatics method make it possible to identify and quantify circRNAs in samples of interest accurately, gaining more insights of the expression dynamics and biological functions of circRNAs. Since circRNAs are non-polyadenylated, poly (A)-selected RNA-Seq data cannot be used for circRNA discovery. Now RNA-Seq is wildly constructed in the study of circRNAs. For example, Ahmed et al. [104] performed paired-end RNA sequencing of primary sites, peritoneal and lymph node metastases from three patients with stage IIIC ovarian cancer and revealed that a significantly larger number of circRNAs were differentially expressed between tumor sites than mRNAs and had a more robust expression pattern than mRNA forms. Thus circRNAs may be more suitable as biomarkers for cancer treatment and prognosis. Hsiao et al.[105] used RNA-Seq data from matched normal and tumor colon tissue samples to identify numerous circRNAs and CircCCDC66 was found to be significantly elevated in polyps and colon cancer, which promoted colon cancer progression, metastasis and poor prognosis. Recently, Yao et al. [106] demonstrated ZKSCAN1, which is expressed in both linear and circular (circZKSCAN1) forms of RNA in human HCC tissues and cell lines, was significantly lower in the HCC samples compared with that in matched adjacent non-tumorous tissues. But RNA-seq revealed that ZKSCAN1 mRNA mainly regulated cellular metabolism, while circZKSCAN1 mediated several cancer-related signaling pathways, including PI3K pathway, migration pathway, actin cytoskeleton pathway, adhesion pathway and cytokine interaction pathway, suggesting ZKSCAN1 mRNA and circZKSCAN1 may cooperate closely with one another to inhibit growth, migration, and invasion of HCC. Furthermore, cirZKSCAN1 might be a useful marker for the diagnosis of HCC.

## CONCLUSIONS

It has been more than two decades since circRNAs were first discovered. Though circRNAs were originally thought to be splicing mistakes, but with the development of high-throughput sequencing technologies and bioinformatics method, circRNAs are increasingly investigated. In this review, we first discuss about the origins, characteristics and main functions of circRNAs. Based on the function as miRNA sponges, translation and expression regulators and RBP sponges, circRNAs play an important role in cancer and we talk about current research methods of circRNAs in cancer, providing evidence for the significance of circRNAs in cancer diagnosis and clinical treatment.

### Perspective

With the advancement of study method, the investigations of circRNAs are drawing more and more attention. The functions of circRNAs as miRNA sponges, transcriptional regulators and RBP sponges are increasingly recognized and the roles of circRNAs in biogenesis and functional mechanisms in numerous diseases including cancers are becoming the focus of study. These findings elucidate the physiological and pathological processes of a number of circRNAs which may be regarded as novel diagnostic biomarkers and potential therapeutic targets in cancer, but there remain vast majorities of circRNAs to be investigated and specific molecular mechanisms to be explored. Compared with long noncoding RNAs (lncRNAs) and miRNA, circRNAs has the advantage of high stability, which can be regarded as promising clinical biomarkers for diagnosis, prognosis and therapy. In addition, since the first report of abundant presence of circRNAs in exosomes, the studies of circRNAs are coming into a new sight [87]. Exosomes are small membrane vesicles of endocytic origin secreted by most cell types [97]. Reports showed that exosomal circRNAs may be regulated by changes of associated miRNA levels in producer cells and may transfer biological activity to recipient cells. Exosomal circRNAs are likely to be regarded as a new class of exosome-based cancer biomarkers and to play potential biological function. In future studies, with scientists’ endeavors and applications of new approaches, many more circRNAs will be well identified and potential mechanisms will be disclosed to improve the diagnosis and treatment of circRNA-related diseases.

## SUPPLEMENTARY MATERIALS

Summary

Supplementary Table 1

Supplementary Table 2
