# Supplementary material for: Circular RNAs: A novel type of biomarker and genetic tools in cancer
Source: Oncotarget. 2017 Jun 2;8(38):64551–63. doi: 10.18632/oncotarget.18350 (PMC5610025; doi:10.18632/oncotarget.18350)
Supplement: Supplementary file 1 — Summary [file oncotarget-08-64551-s001.pdf]

Circular RNAs: A novel type of biomarker and genetic tools in cancer

Supplementary Materials

Supplementary Table 1: Summary of circRNA databases. See Supplementary_Table_1

Supplementary Table 2: Summary of cancer-associated circRNAs. See Supplementary_Table_2
