# Supplementary material for: Circular RNAs: A novel type of biomarker and genetic tools in cancer
Source: Oncotarget. 2017 Jun 2;8(38):64551–63. doi: 10.18632/oncotarget.18350 (PMC5610025; doi:10.18632/oncotarget.18350)
Supplement: Supplementary file 2 — Supplementary Table 1 [file oncotarget-08-64551-st001.docx]

| **Supplementary Table 1 Summary of circRNA databases** | | | | | | | | |
| --- | --- | --- | --- | --- | --- | --- | --- | --- |
| database | website | Annotation | naming | sample | CircRNA | Associated diseases | profiles of circRNA expression | miRNA |
|  |  |  |  | source | position on |  |  | regulatory |
|  |  |  |  |  | genome |  |  | relationships |
| circ2Traits ^[19]^ | http://  gyanxet-beta.  com/circdb/ | 1953 Human  circRNAs | A serial number  for every  detected  back-spliced  junction site | Not available | A customised  genome browser | 105 Diseases | Not available | Identify miRNA and  circRNA  interactions |
| circBase ^[20]^ | http://www.  circbase.org/ | Not available | A serial number  for every  detected  back-spliced  junction site | The samples  where the  back-spliced  junction sites  were discovered | Linked out to  UCSC Genome  Browser | Not available | Not available | Not available |
|  |  |  |  |  |  |  |  |  |
| CircNet ^[21]^ | http://circnet.mbc.nctu.  edu.tw/ | 212 950 CircRNAs | A systematic naming  system that provides  information to the source gene and  annotated exons of  circRNAs | 1. Expression  level in available  samples.  2. In which sample  junction sites were  discovered.  3. Clustered  sample conditions | An integrated genome  browser synchronising  with the network  graphical user interface | Not available | An all-sample expression  heat-map for every circRNA and linear  isoform | the relationship  between miRNA target genes and circRNAs shown by a network-driven  graphical interface |
| CircInteractome ^[22]^ | http://circinteractome.  nia.nih.gov | Not available | Same as circBase | Not available | Linked out to UCSC  genome browser | Not available | Not available | Identify circRNA  and miRNA  interactions |
| circRNADb ^[23]^ | <http://reprod.njmu.edu.cn/circrnadb> | 32,914 human exonic circRNAs | Not available | Not available | Not available | Not available | Not available | Identify circRNA and miRNA interactions |
| deepBase v2.0 ^[24]^ | http://biocenter.  sysu.edu.cn/  deepBase/ | 14 867 Human  circRNAs | A systematic  naming system  that provides  information to the  transcript number | Not available | A customised  genome browser | Not available | Not available | Not available |
| nc2Cancer ^[25]^ | http://www.  bioinfo.tsinghua.  edu.cn/nc2Cancer | 172 Human  circRNAs | A serial number  for every detected  back-spliced | Not available | Not available | 31 Cancers | Not available | Identifies circRNA  and miRNA  interactions |
|  | http://starbase.  sysu.edu.cn | Not available | junction site  same as circBase,  except CDR1  antisense  (CDR1as) | circBase v0.1  source samples | A customised  genome browser  accessible  through keyword  search | Not available | Not available | Identify circRNA  and miRNA  interactions  through Chip-Seq  data analysis |
| starBase v2.0 ^[26]^ |  |  |  |  |  |  |  |  |
