# Supplementary material for: Circular RNAs: A novel type of biomarker and genetic tools in cancer
Source: Oncotarget. 2017 Jun 2;8(38):64551–63. doi: 10.18632/oncotarget.18350 (PMC5610025; doi:10.18632/oncotarget.18350)
Supplement: Supplementary file 3 — Supplementary Table 2 [file oncotarget-08-64551-st002.docx]

| **Supplementary Table 2 Summary of cancer-associated circRNAs** | | | |
| --- | --- | --- | --- |
| CircRNAs | Expression | Function | Cancer |
| CDR1as/ciRS-7* | Up-regulated | miR-7 sponge | Astrocytoma^[41]^ |
|  |  |  | Hepatocellular carcinoma^[42]^ |
|  |  |  | Gastric carcinoma^[43]^ |
|  |  |  | Colorectal cancer^[44]^ |
|  |  |  | Breast cancer^[45]^ |
|  |  |  | Cervical cancer^[46]^ |
|  |  |  | Lung carcinomas^[26]^ |
|  |  |  | Tongue cancer^[47]^ |
|  |  |  | Schwannoma tumor^[48]^ |
| cir-ITCH* | Down-regulated | miR-7,miR-20a sponges | Colorectal cancer^[59]^ |
|  |  | miR-7,miR-17, miR-214 sponges |  |
|  |  | miR-7,miR-214 sponges | Esophageal squamous cell carcinoma^[12]^ |
|  |  |  | Lung cancer^[60]^ |
|  |  |  |  |
| circHIPK3* | Uncertain | Multiple miRNAs sponges, especially miR-124 | Several cancers^[61]^ |
| circ-Foxo3* | Down-regulated | Enhance Foxo3 expression, form circ-Foxo3-p21-CDK2 ternary complex | Breast cancer^[62,77]^  Colorectal cancer ^[78]^  Non-small cell lung cancernon-small cell lung cancer ^[79]^ |
| circRNA_1093* | Up-regulated | miR-342-3p sponge | Breast cancer^[63,64]^ |
|  |  |  |  |
| circ-ZEB1.17* circ-ZEB1.19* | Down-regulated | miR-200 sponge | Lung cancer^[21]^ |
| circ-ZEB1.33* |  |  |  |
| circ-ZEB1.5* |  |  |  |
| hsa_circ_001569 | Up-regulated | miR-145 sponge | colorectal cancer^[13]^ |
| circ-TTBK2* | Up-regulated | miR-217 sponge | Glioma^[65]^ |
| ci-mcm5* | Up-regulated | Enhance MCM5 | Colorectal cancer^[67]^ |
|  |  | expression | Oral squamous cell carcinoma^[68]^ |
|  |  |  |  |
| ci-sirt7* | Down-regulated | Enhance SIRT7 expression | Pancreatic ductal adenocarcinoma^[69]^ |
| cZNF292* | Down-regulated | inhibite tube formation and vascularization | Glioma^[70]^ |
| hsa_circ_0031288* | Up-regulated | Competitively suppresse HuR binding to PABPN1 mRNA | Cervical carcinoma^[80]^ |
| hsa_circ_100855* | Up-regulated | potential biomarker | laryngeal cancer^[81]^ |
| hsa_circ_104912* | Down-regulated | potential biomarker | laryngeal cancer^[81]^ |
| hsa_circ_002059* | Down-regulated | Potential biomarker | Gastric cancer^[14]^ |
| hsa_circ_0000190* | Down-regulated | Potential biomarker | Gastric cancer^[82]^ |
| hsa_circ_0000096* | Down-regulated | Potential biomarker | Gastric cancer^[83]^ |
| CircPVT1* | Up-regulated | Potential biomarker | Gastric cancer^[84]^ |
| hsa_circ_0001649* | Down-regulated | Potential biomarker | Hepatocellular carcinoma^[85]^ |
| hsa_circ_0005075* | Up-regulated | Potential biomarker | Hepatocellular carcinoma^[86]^ |
| hsa_circ_001988* | Down-regulated | Potential biomarker | Colorectal cancer^[87]^ |
| hsa_circ_0000069* | Up-regulated | Potential biomarker | Colorectal cancer^[88]^ |
| circ-BANP* | Up-regulated | Potential biomarker | Colorectal cancer^[89]^ |
| circBRAF* | Down-regulated | Potential biomarker | Glioma^[92]^ |
| hsa_circ_0067934* | Up-regulated | potential biomarker | Esophageal squamous cell carcinoma^[93]^ |
| circTCF25* | Up-regulated | potential biomarker | Bladder cancer^[94]^ |
| f-circM9*  f-circPR* | Up-regulated | potential biomarker | leukemia^[97]^ |
| hsa_circ_001059^#^ hsa_circ_000167^#^ | Up-regulated | _ | esophageal cancer^[100]^ |
| circRNA MYL^#^ | Up-regulated | enhance DNMT3B, VEGFA and ITGB1 expressions | Bladder cancer^[101]^ |
| CircCCDC66* | Up-regulated | _ | colon cancer^[105]^ |
| circZKSCAN1* | Down-regulated | mediate PI3K pathway, migration pathway, actin cytoskeleton pathway, adhesion pathway and cytokine interaction pathway | hepatocellular carcinoma^[106]^ |

*means the circRNAs has been experimentally validated.

^#^means the circRNAs are proposed by bioinformatics predication.
